# Does Human Experimental Endotoxemia Impact Negative Cognitions Related to the Self?

**DOI:** 10.3389/fnbeh.2018.00183

**Published:** 2018-08-21

**Authors:** Simone Kotulla, Sigrid Elsenbruch, Till Roderigo, Alexandra Brinkhoff, Alexander Wegner, Harald Engler, Manfred Schedlowski, Sven Benson

**Affiliations:** ^1^Institute of Medical Psychology and Behavioral Immunobiology, University Hospital Essen, University of Duisburg-Essen, Essen, Germany; ^2^Department of Nephrology, University Hospital Essen, University of Duisburg-Essen, Essen, Germany; ^3^Clinic for Trauma and Orthopedic Surgery, University Hospital Essen, University of Duisburg-Essen, Essen, Germany

**Keywords:** systemic inflammation, lipopolysaccharide, cytokines, TNF-α, depression, mood, hopelessness, self-esteem

## Abstract

A role of inflammatory processes in the pathophysiology of depression is increasingly recognized. Experimental endotoxemia offers an established model to induce transient systemic inflammation in healthy humans, and has been proposed as an experimental paradigm of depression. Indeed, different symptoms of depression can be observed during experimental endotoxemia, including negative mood or dysthymia as key symptoms of depression. Hopelessness and low self-esteem constitute common cognitive symptoms in depression, but have not been specifically assessed during endotoxemia. Thus, we pooled data from healthy volunteers who received low-dose endotoxin (i.e., 0.4 or 0.8 ng/kg lipopolysaccharide, LPS) or placebo in three randomized, controlled studies to investigate the effects of LPS on cognitive schemata related to depression. Validated questionnaires were used to assess self-esteem, hopelessness and the vulnerability factor intolerance of uncertainty after intravenous injection of LPS or placebo. Plasma tumor necrosis factor (TNF)-α and interleukin (IL)-6 were repeatedly assessed, along with self-reported mood. Because not all questionnaires were available from primary studies, data were analyzed in two separate data sets: In data set 1, self-esteem and intolerance of uncertainty were assessed in *N* = 87 healthy volunteers, who randomly received either 0.4 or 0.8 ng/kg LPS or placebo. In data set 2, hopelessness was measured in *N* = 59 volunteers who randomly received either LPS (0.8 ng/kg) or placebo. In both data sets, LPS-application led to significant increases in TNF-α and IL-6, reflecting systemic inflammation. Positive mood was significantly decreased in response to LPS, in line with inflammation-induced mood impairment. General self-esteem, intolerance of uncertainty and hopelessness did not differ between LPS- and placebo groups, suggesting that these negative cognitive schemata are not responsive to acute LPS-induced systemic inflammation. Interestingly, LPS-treated volunteers reported significantly lower body-related self-esteem, which was associated with increased TNF-α concentration. Thus, certain aspects of self-esteem related to physical attractiveness and sportiness were reduced. It is conceivable that this effect is primarily related to physical sickness symptoms and reduced physical ability during experimental endotoxemia. With respect to cognitive symptoms of depression, it is conceivable that LPS affects cognitive *processes*, but not negative cognitive schemata, which are rather based on learning and repeated experiences.

## Introduction

Major depression (MD) is a prevalent and severe psychiatric disorder characterized by depressed mood, loss of interest or pleasure, changes in weight, appetite, sleep and activity, fatigue and suicidality (Kessler et al., 2005; American Psychiatric Association, 2013; Hasin et al., 2018). Another key feature of depression is neurocognitive symptoms. These not only include changes in information processing, impaired concentration and indecisiveness, but also negative cognitions related to the self, the past, present and future, contributing to feelings of worthlessness, hopelessness and excessive or inappropriate guilt (Clark and Beck, 2010; American Psychiatric Association, 2013; Uher et al., 2014), and ultimately to depression onset and maintenance (Evans et al., 2005; Disner et al., 2011). The pathogenesis of MD remains incompletely understood, which hampers the development of new therapeutic approaches (DellaGioia and Hannestad, 2010).

A role of inflammatory processes in MD pathophysiology is increasingly recognized (Capuron and Miller, 2004; DellaGioia and Hannestad, 2010; Kiecolt-Glaser et al., 2015; Yirmiya et al., 2015; Miller and Raison, 2016; Otte et al., 2016), offering treatment perspectives for at least a subgroup of patients. Anti-inflammatory treatment reportedly improves symptoms in patients resistant to classical anti-depressant medication (Tyring et al., 2006; Miller and Raison, 2016). Elevated inflammatory markers, such as C-reactive protein (CRP) and the pro-inflammatory cytokine interleukin (IL)-6 have been demonstrated in a substantial proportion of MD patients (Howren et al., 2009; DellaGioia and Hannestad, 2010; Dowlati et al., 2010). In depression-free individuals, increased concentrations of inflammatory markers can independently predict the incidence of depression over a time period of 10 or more years (Gimeno et al., 2009; Pasco et al., 2010; Raison and Miller, 2011). Finally, MD is more prevalent in patients with chronic inflammation (Patten et al., 2003; Graff et al., 2009; Matcham et al., 2013). It remains however unclear if inflammatory processes are related to all symptom domains of MD or may rather be associated with only specific symptoms, calling for preclinical research.

The experimental administration of low-dose endotoxin (e.g., lipopolysaccharide, LPS) constitutes an established translational model to analyze the effects of systemic inflammation on mood, cognition and behavior (Andreasen et al., 2008; Schedlowski et al., 2014; Suffredini and Noveck, 2014; Lasselin et al., 2018). LPS is a cell-wall component of Gram-negative bacteria that activates the innate immune system through a Toll-like receptor 4-dependent pathway, ultimately leading to a systemic increase in pro-inflammatory cytokines (Andreasen et al., 2008; Schedlowski et al., 2014; Zouikr and Karshikoff, 2017). Via vagal and humoral afferent pathways, these cytokines act on the brain and induce changes in mood and behavior, which are commonly referred to as sickness behavior (Dantzer and Kelley, 2007; Schedlowski et al., 2014). Sickness behavior shows striking similarities to several key symptoms of depression, although sickness behavior symptoms are transient and typically disappear when inflammation has resolved (Dantzer et al., 2008; Raison and Miller, 2011). Low doses of endotoxin reliably induce transient symptoms including negative mood (Reichenberg et al., 2001; Wright et al., 2005; Eisenberger et al., 2009, 2010b; Hannestad et al., 2011; Benson et al., 2017a; Engler et al., 2017), feelings of social isolation and disturbed psychosocial functioning (Eisenberger et al., 2009, 2010b; Inagaki et al., 2015; Moieni et al., 2015a,b), changes in motivation (Eisenberger et al., 2010a; Lasselin et al., 2017) and fatigue (Benson et al., 2017b).

Whether experimental endotoxemia also impacts neurocognitive processes relevant to the characteristic negative cognitive schemata of worthlessness, low self-esteem, guilt and hopelessness in MD remains unclear. It has been proposed that these symptoms may not change in response to LPS (DellaGioia and Hannestad, 2010), however first findings (Eisenberger et al., 2009) showed LPS-induced changes in the Profile of Mood States (POMS) depression scale, a subscale which comprises items related to worthlessness and hopelessness. Further, we recently documented a negative cognitive bias, i.e., a prolonged and more sustained processing of negative information during low-dose endotoxemia (Benson et al., 2017a). This effect was only seen when sad mood was experimentally induced during systemic inflammation using a mood induction paradigm (Benson et al., 2017a). Together, these findings support the notion that systemic inflammation not only increases the susceptibility to negative emotional stimuli, but also changes the cognitive processing of negative information (Bollen et al., 2017). However, this is indirect evidence, and no dedicated data exist thus far in the experimental human endotoxemia literature to address negative cognitive schemata. We herein aim to close this research gap by exploring effects of LPS on self-esteem, hopelessness, and intolerance of uncertainty. Intolerance of uncertainty reflects the tendency to consider possible negative events as frightening or burdening, and constitutes a vulnerability factor for depression and anxiety disorders (McEvoy and Mahoney, 2011; Carleton et al., 2012; Boelen and Lenferink, 2018; Lauriola et al., 2018). Symptoms were assessed with validated questionnaires in a comparatively large cohort of healthy volunteers who participated in double-blind, placebo-controlled LPS studies.

## Materials and Methods

### Participants

Data from a total of *N* = 109 healthy volunteers (89 men, 20 women) who were randomized to receive either low-dose LPS (0.4 or 0.8 ng/kg body weight) or saline (placebo) were analyzed. All volunteers participated in one of three randomized, double-blind endotoxemia studies (Wegner et al., 2015; Benson et al., 2017a, unpublished data), and completed validated questionnaires to analyze negative cognitive schemata related to depression, along with changes in mood. For cross-over studies (Wegner et al., 2015; Benson et al., 2017a), only data from the first study day was included to avoid carry-over effects (i.e., data from the second day were discarded for the purposes of this analysis). Identical methods were used to measure cytokine concentrations and mood, which allows the pooling of data across studies. Questionnaire data on self-esteem and intolerance of uncertainty were available from two studies with a total of *N* = 87 participants (Wegner et al., 2015; Benson et al., unpublished data), and were pooled for analysis to *data set 1*. Hopelessness was measured in *N* = 59 volunteers (Benson et al., 2017a unpublished data), and data were pooled to *data set 2*. All studies were conducted in accordance with the Declaration of Helsinki and were approved by the Institutional Ethics Review Board of the Medical Faculty of the University of Duisburg-Essen (permit numbers: 09-4271; 15-6234-BO; 15-6533-BO). All participants provided written informed consent and received financial compensation for study participation.

### Recruitment and Safety Routine

For all primary studies, recruitment, inclusion and exclusion criteria and safety routine were identical and have previously been reported in detail (Wegner et al., 2015; Benson et al., 2017a). Briefly, healthy volunteers aged 18–45 years were recruited via public announcements and underwent an in-depth screening procedure consisting of a structured telephone interview, a physical examination and personal interview conducted by a physician, and repeated laboratory assessments (blood cell count, liver enzymes, renal parameters, electrolytes, coagulation factors and CRP). General exclusion criteria were any pre-existing or current physical or psychiatric illness, pregnancy, body mass index (BMI) <18 or ≥29 kg/m^2^, current medications, smoking or regular alcohol use (>4 drinks per week). Female participants were only included when taking oral contraceptives to prevent confounding effects of menstrual cycle phase. Pregnancy was ruled out with a urinary pregnancy test on the study day. A diagnosis of depression or clinically-relevant depression symptoms exceeding published cut-off scores of the Beck Depression Inventory (BDI; Hautzinger et al., 1995) were exclusionary for ethical reasons, and also to avoid confounding effects on primary outcome variables, i.e., cognitive symptoms of depression. Participants were told to refrain from strenuous exercise 48 h prior to study days. Safety measures included monitoring for at least 6 h and follow-up examinations 24 h and 7 days after the injection of LPS or placebo.

### Study Protocol

*Data set 1* consisted of *N* = 87 healthy male and female volunteers who were randomized to receive either 0.4 ng/kg (*N* = 29) or 0.8 (*N* = 20) ng/kg body weight LPS or placebo (*N* = 38). *Data set 1* includes data from *N* = 20 female volunteers who received either 0.4 ng/kg body weight LPS (*n* = 10) or placebo (*n* = 10). *Data set 2* comprised data from *N* = 59 healthy men who received either 0.8 ng/kg body weight LPS (*N* = 25) or placebo (*N* = 34). LPS (reference standard endotoxin from *Escherichia coli*, serotype O113:H10:K-negative, lot H0K354, United States Pharmacopeia, Rockville, MD, USA; LPS conditions) or saline (placebo condition) was injected via an intravenous catheter placed in an antecubital forearm vein. Blood samples for cytokine analyses were collected before (baseline, BL) as well as 1, 2, 3, and 6 h after injection of LPS or placebo. Body temperature (with an intra-aural thermometer), blood pressure, and heart rate were assessed after blood sampling. Changes in self-reported mood (positive vs. negative) were assessed with the respective subscale of the standardized and validated German multidimensional mood questionnaire (MDBF; Steyer et al., 1997) before (BL) as well as 3 and 6 h after injection of LPS or placebo. Cognitive symptoms of depression were assessed with three validated questionnaires (see below) 3 h post injection. This time point was chosen for two reasons: First, increased plasma concentrations of tumor necrosis factor (TNF)-α and IL-6 were consistently observed 2–4 h post injection in previous studies, indicating systemic immune activation (Wegner et al., 2014; Benson et al., 2017b). More importantly, we found a significant rise in IL-6 in the cerebrospinal fluid 3 h post-injection (Engler et al., 2017), reflecting a CNS response to peripheral inflammation.

#### Cognitive Symptoms of Depression

##### Self-Esteem/Self-Worth (Data Set 1)

Self-esteem was assessed with the Multidimensional Self-Worth Scale (MSWS; Schütz et al., 2006), a German adaption of the Multidimensional Self-Concept Scale (MSCS; Fleming and Courtney, 1984). The MSWS contains 32 seven-point Likert-scaled items. The MSWS consists of six subscales, which can be combined to a “general self-esteem” (AWS) scale and a “body-related self-esteem” (KSW) scale. General self-esteem comprises aspects of “emotional,” “social,” “conflict-related,” and “performance-related self-esteem,” and body-related self-esteem refers to “self-regarded physical attractiveness” and “self-regarded sportiness.” The reliability for the “general self-esteem” (AWS) scale (Cronbach’s α = 0.92) and “body-related self-esteem” (KSW) scale (Cronbach’s α = 0.85) can be consider as good based on internal reliability (Cronbach’s α, Schütz et al., 2006).

##### Intolerance of Uncertainty (Data Set 1)

Intolerance of uncertainty reflects the tendency to consider possible negative events as frightening or burdening, and constitutes a vulnerability factor for depression and anxiety disorders (McEvoy and Mahoney, 2011; Carleton et al., 2012; Boelen and Lenferink, 2018; Lauriola et al., 2018). Intolerance of uncertainty was assessed with the validated German UI-18 (“*Unsicherheitsinteroleranz-Skala*,” Gerlach et al., 2008) questionnaire, an adaption of the Intolerance of Uncertainty Scale (Freeston et al., 1994). The UI-18 contains 18 items which are answered on a 5-point-Likert-scale, and can be combined to the three subscales “reduced ability to act due to intolerance of uncertainty,” “burden due to intolerance of uncertainty” and “vigilance due to intolerance of uncertainty” (Gerlach et al., 2008). All scales assess how people react on uncertainties of life. The scales “burden” and “vigilance” were shown to reliably predict worrying (Gerlach et al., 2008). Reliability for subscales (Cronbach’s α ≥ 0.80), and the overall internal reliability (Cronbach’s α = 0.90) can be considered as good (Gerlach et al., 2008).

##### Hopelessness (Data Set 2)

Hopelessness was measured with the revised version of the validated German Hopelessness-scales (H-R-scale; *Skalen zur Erfassung von Hoffnungslosigkeit*; Krampen, 2004). The H-R-scale was theoretically based on Beckś cognitive theory of depression (Beck et al., 1979). The scale assesses the tendency to evaluate the future in negative terms, and therefore reflects a pessimistic cognitive style. The H-R-scale contains 20 six-point Likert-scaled items. Sufficient to good internal consistency has been reported (Cronbachs α = 0.74—α = 0.92) for different reference samples).

### Plasma Cytokine Concentrations, Leukocyte Counts and C-Reactive Protein

Blood for cytokine analyses was collected in EDTA-treated tubes (S-Monovette, Sarstedt, Nümbrecht, Germany). Plasma was immediately separated by centrifugation (2,000 *g*, 10 min, 4°C) and stored at −80°C until analysis. Plasma TNF-α and IL-6 concentrations were measured using enzyme-linked immunosorbent assays (ELISA; Human Quantikine ELISA, R&D Systems, Minneapolis, MN, USA) according to the manufacturer’s protocols. The sensitivity of the assays was 0.11 pg/ml for TNF-α and 0.70 pg/ml for IL-6. Mean inter- and intra-assay coefficients of variation were ≤10%. In data set 1, one TNF-α and three IL-6 samples were below the respective detection limits, as well as in data set 2 two TNF-α samples and one IL-6 sample. White blood cell (WBC) counts were determined using an automated cell counter (Sysmex KX-21N, Norderstedt, Germany). CRP concentration was measured before and 24 h post-injection with a polyethylene glycol (PEG)-enhanced immunoturbidimetric assay (sensitivity 0.5 mg/dl) by the Division of Laboratory Research of the University Hospital Essen (Germany).

### Statistical Analyses

Data analysis was performed using SPSS 22.0 (SPSS Inc., Chicago, IL, USA) and the level of significance was set at *α* < 0.05. Normal distribution was tested using Kolmogorov-Smirnov-test, and non-normally distributed variables (i.e., plasma cytokines) were log-transformed before analysis. Data are shown as mean ± standard error of the mean (SEM). BL sociodemographic and psychological parameters of LPS and placebo groups were compared with univariate analysis of variance (ANOVA; data set 1) or unpaired *t*-tests (data set 2). LPS effects on plasma cytokines, body temperature and mood were analyzed in both data sets with repeated measures ANOVA (rm-ANOVA) with endotoxin condition as group (LPS vs. placebo) and time as within-subject factor. Greenhouse-Geisser correction was applied where appropriate. Bonferroni-corrected *post hoc* unpaired *t*-tests (two-tailed) were computed in case of significant rm-ANOVA interaction effects. To assess group differences in cognitive symptoms of depression (i.e., self-esteem, intolerance of uncertainty, hopelessness) analyses of covariance (ANCOVA) were computed. To exclude that effects were confounded by inter-individual differences in BL depressive symptoms, BL BDI scores were entered as a covariate in these analyses. As an indicator of effect sizes, partial Eta^2^ ($\eta_{\text{p}}^{2}$) was computed for ANOVA/ANCOVA. Partial Eta^2^ allows to estimate effect sizes within the relevant population as it indicates the percent of variance of measures (e.g., self-esteem scores), which is explained by a respective factor (e.g., LPS vs. placebo; Fields, 2013). Correlations were computed as Pearson’s *r*.

## Results

### Sample Characteristics

*Data set 1* comprised *N* = 87 healthy volunteers (20 women) with a mean age of 26.9 ± 0.1 years and a mean BMI of 23.6 ± 0.3 kg/m^2^. *Data set 2* consisted of *N* = 59 healthy men with a mean age of 26.5 ± 0.6 years and mean BMI of 24.1 ± 0.3 kg/m^2^. No differences in age, BMI or BL BDI scores were observed between LPS- and placebo groups (Table 1).

**Table 1:** Sociodemographic and psychological characteristics for data set 1 (upper part) and data set 2 (lower part).

Data set 1	Placebo	0.4 ng/kg LPS	0.8 ng/kg LPS	Test statistic	*P*
	(*N* = 38)	(*N* = 29)	(*N* = 20)		
Age (years)	26.71 ± 1.33	27.24 ± 1.40	26.80 ± 1.07	*F* = 0.16	0.89
Body mass index (kg/m^2^)	23.92 ± 0.70	23.08 ± 0.72	23.52 ± 0.57	*F* = 0.89	0.41
Sex (*N*)	*m* = 28, *f* = 10	*m* = 19, *f* = 10	*m* = 20, *f* = 0	/	/
BDI score	3.18 ± 0.87	2.93 ± 0.91	2.90 ± 0.70	*F* = 0.07	0.92
**Data set 2**	**Placebo**		**0.8 ng/kg LPS**	**Test statistic**	***P***
	**(*N* = 34)**		**(*N* = 25)**		
Age (years)	26.02 ± 0.96		27.12 ± 0.77	*t* = −0.83	0.40
Body mass index (kg/m^2^)	24.32 ± 0.38		23.67 ± 0.47	*t* = 1.07	0.28
BDI score	3.11 ± 0.53		2.64 ± 0.62	*t* = 0.58	0.56

*BDI, Beck Depression Inventory. All data are shown as mean ± SEM unless otherwise indicated. m = male, f = female. *P* = *P*-value for results of univariate ANOVA (data set 1) or independent samples t-tests (data set 2)*.

### Plasma Cytokines and Body Temperature

**Figure 1:**
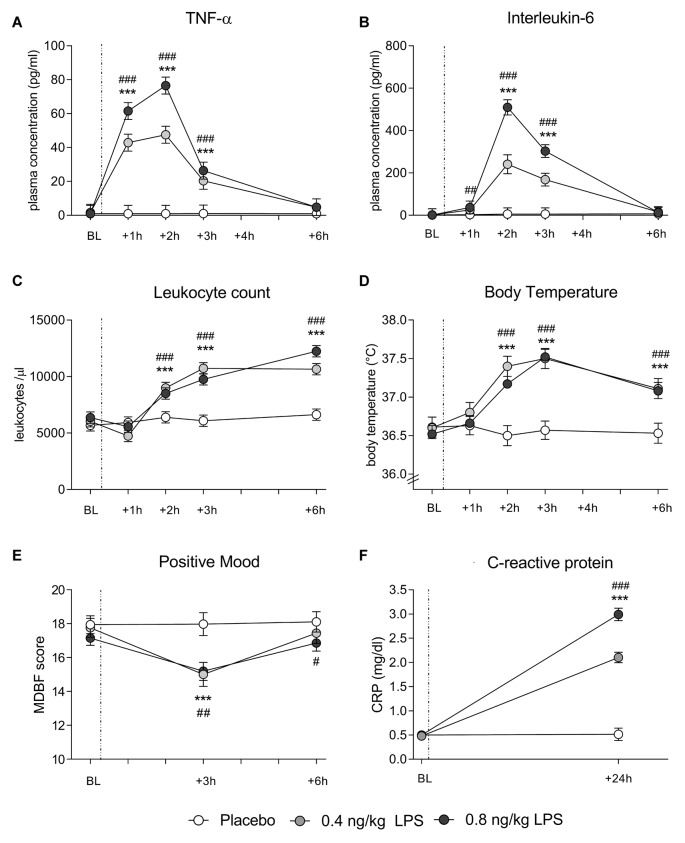
Data set 1: plasma concentrations of tumor-necrosis-factor (TNF)-α **(A)** and Interleukin (IL)-6 **(B)** leukocyte counts **(C)** and body temperature **(D)** were measured before (baseline, BL), and 1, 2, 3 and 6 h after injection of either 0.8 ng/kg body weight lipopolysaccharide (LPS; black dots), 0.4 ng/kg body weight LPS (gray dots), or saline (placebo group; white dots). Mood **(E)** was assessed with the respective subscale of the German Multi-Dimensional Mood (MDBF) questionnaire at BL, as well as 3 and 6 h after injection. C-reactive protein (CRP) **(F)** was measured at BL and 24 h post injection. ^###^*p* < 0.001, ^##^*p* < 0.01, ^#^*p* < 0.05 0.8 ng/kg LPS group vs. placebo group. ***p < 0.001, 0.4 ng/kg LPS group vs. placebo group. For results of repeated measures analysis of variance (ANOVA), see text.

**Figure 2:**
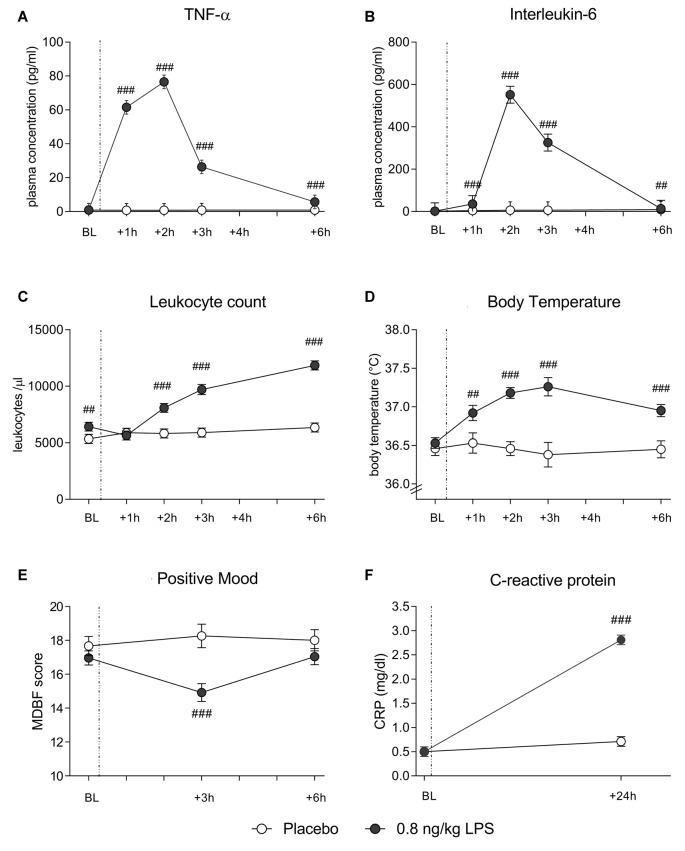
Data set 2: plasma concentrations of TNF-α **(A)** and IL-6 **(B)**, leukocyte counts **(C)** and body temperature **(D)** were measured before (BL), and 1, 2, 3 and 6 h after injection of either 0.8 ng/kg body weight LPS (black dots) or saline (placebo group; white dots). Mood **(E)** was assessed with the respective subscale of the German Multi-Dimensional Mood (MDBF) questionnaire at BL, as well as 3 and 6 h after injection. CRP **(F)** was measured at BL and 24 h post injection. ^###^*p* < 0.001, ^##^*p* < 0.01, 0.8 ng/kg LPS group vs. placebo group. For results of repeated measures ANOVA, see text.

In both data sets, LPS administration expectedly led to transient increases in plasma cytokine and CRP concentrations, leukocyte counts and body temperature (see Figure 1 for data set 1 and Figure 2 for data set 2). Specifically, LPS application induced a significant increase in plasma concentrations of TNF-α (*data set 1*: *F* = 29.4, *p* < 0.001, $\eta_{\text{p}}^{2}$ = 0.41; *data set 2*: *F* = 60.0, *p* < 0.001, $\eta_{\text{p}}^{2}$ = 0.62) and IL-6 (*data set 1*: *F* = 47.7, *p* < 0.001, $\eta_{\text{p}}^{2}$ = 0.54; *data set 2*: *F* = 80.5, *p* < 0.001, $\eta_{\text{p}}^{2}$ = 0.59), along with a significant rise in circulating leukocyte numbers (*data set 1*: *F* = 29.4, *p* < 0.001, $\eta_{\text{p}}^{2}$ = 0.42; *data set 2*: *F* = 44.5, *p* < 0.001, $\eta_{\text{p}}^{2}$ = 0.44), body temperature (*data set 1*: *F* = 14.1, *p* < 0.001, $\eta_{\text{p}}^{2}$ = 0.25; *data set 2*: *F* = 10.9, *p* < 0.001, $\eta_{\text{p}}^{2}$ = 0.16), and CRP concentrations (*data set 1*: *F* = 122.1, *p* < 0.001, $\eta_{\text{p}}^{2}$ = 0.75; *data set 2*: *F* = 121.8, *p* < 0.001, $\eta_{\text{p}}^{2}$ = 0.69; all ANOVA interaction effects). *Post hoc* Bonferroni-tests revealed significant differences between LPS groups and placebo groups in both data sets, but not between 0.4 and 0.8 ng/kg LPS groups (for results of *post hoc* tests, see Figures 1, 2).

### LPS Effects on Mood

LPS administration led to a transient decline in positive mood in response to LPS (*data set 1*: *F* = 8.4, *p* < 0.001, $\eta_{\text{p}}^{2}$ = 0.16; *data set 2*: *F* = 10.7, *p* < 0.001, $\eta_{\text{p}}^{2}$ = 0.15; ANOVA interaction effects). *Post hoc* Bonferroni tests supported that mood was significantly impaired in the LPS groups when compared to the placebo groups in both data sets. No differences were found between 0.4 and 0.8 ng/kg LPS groups in data set 1 (for results of *post hoc* tests, see Figures 1, 2).

### LPS Effects on Cognitive Symptoms of Depression

#### Self-Esteem (Data Set 1)

**Figure 3:**
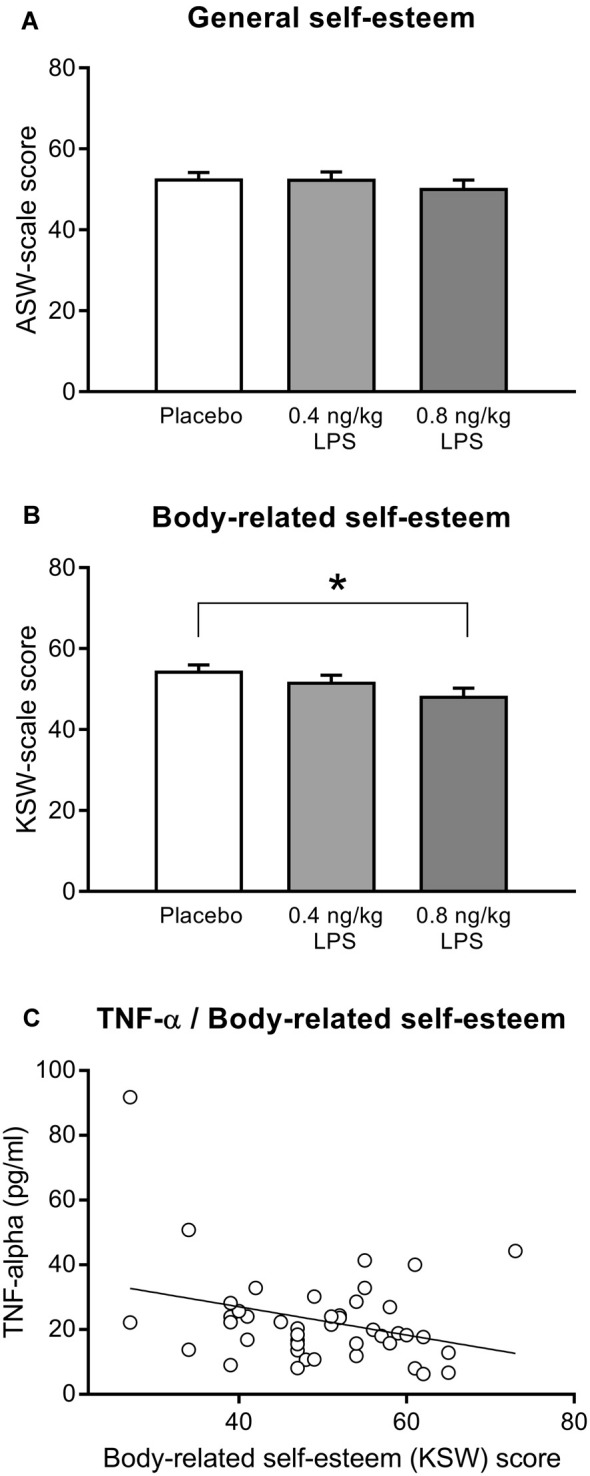
General self-esteem **(A)** and body-related self-esteem **(B)** were assessed with the validated German Multidimensional Self-Worth Scale (MSWS) questionnaire (see text for details) 3 h after injection of either 0.8 or 0.4 ng/kg body weight LPS, or saline (placebo group). Exploratory correlation analysis within the LPS group **(C)** indicated a significant association between body-related self-esteem and TNF-α plasma concentration 3 h after injection (*r* = −0.31, *p* < 0.05; for details, see text). *p < 0.05, result of Bonferroni-corrected *post hoc* *t*-test. For results of ANOVA, see text.

General (Figure 3A) and body-related (Figure 3B) self-esteem were assessed with the validated MSWS questionnaire. Participants who received LPS reported a significantly lower body-related self-esteem (*F* = 3.57, *p* = 0.03, $\eta_{\text{p}}^{2}$ = 0.08). *Post hoc* testing revealed that the higher LPS group (0.8 ng/kg) displayed significantly lower scores for body-related self-esteem compared to the placebo group (*p* = 0.03), while the lower dose LPS group (0.4 ng/kg) differed neither from the placebo (*p* = 0.60) nor from the 0.8 ng/kg LPS dose group (*p* = 0.49). LPS and placebo groups did not differ in general self-esteem (*F* = 0.55, *p* = 0.57, $\eta_{\text{p}}^{2}$ = 0.01). Exploratory correlation analysis within LPS-treated volunteers revealed a significant association between higher TNF-α concentrations 3 h post injection and lower body-related self-esteem (MSWS-scores; *r* = −0.31, *p* < 0.05, Figure 3C). No further significant association between body-related self-esteem and inflammatory parameters including IL-6 (*r* = −0.23, *p* = 0.13), CRP (*r* = −0.22, *p* = 0.14), and body temperature (*r* = −0.27, *p* = 0.064) were observed.

#### Intolerance of Uncertainty (Data Set 1)

Intolerance of uncertainty was assessed with the validated UI-18 questionnaire (Table 2). No significant group differences were observed for the three subscales “reduced ability to act” (*F* = 1.45, *p* = 0.23, $\eta_{\text{p}}^{2}$ = 0.03), “burden” (*F* = 1.01, *p* = 0.36, $\eta_{\text{p}}^{2}$ = 0.02), or “vigilance” (*F* = 0.22, *p* = 0.79, $\eta_{\text{p}}^{2}$ = 0.01).

**Table 2:** Intolerance of uncertainty (data set 1).

UI-18 subscales	Placebo	0.4 ng/kg LPS	0.8 ng/kg LPS	*F*	*P*
	(*N* = 36)	(*N* = 28)	(*N* = 20)		
Reduced ability to act	10.55 ± 0.94	11.14 ± 0.99	12.00 ± 0.83	1.45	0.23
Burden	13.33 ± 1.16	13.00 ± 1.22	11.55 ± 1.02	1.01	0.36
Vigilance	14.38 ± 1.20	14.00 ± 1.26	13.45 ± 1.06	0.22	0.79

*Intolerance of uncertainty was assessed in data set 1 using the validated UI-18 questionnaire (Gerlach et al., 2008). Data from two participants of the placebo group and one participant from the 0.4 ng/kg LPS group are missing. All data are shown as mean ± SEM. *P* = *P*-value for results of univariate ANCOVA group effect accounting for baseline BDI scores*.

#### Hopelessness (Data Set 2)

Hopelessness was assessed with the validated H-R-scale. Although LPS-treated participants showed slightly higher hopelessness scores (47.2 ± 1.4) compared to the placebo group (46.1 ± 1.8), no significant difference was found (*F* = 0.57, *p* = 0.45, $\eta_{\text{p}}^{2}$ = 0.01).

## Discussion

Inflammatory processes are increasingly recognized in the pathophysiology of MD (Capuron and Miller, 2004; Schiepers et al., 2005; Raison et al., 2006; Kiecolt-Glaser et al., 2015; Yirmiya et al., 2015; Miller and Raison, 2016; Otte et al., 2016). This is supported by experimental and clinical data suggesting that systemic inflammation contributes to an increased risk of depression (Haroon et al., 2012; Kiecolt-Glaser et al., 2015; Miller and Raison, 2016; Otte et al., 2016). Furthermore, it has been demonstrated that the experimental induction of systemic inflammation in healthy volunteers, e.g., by injecting low-doses of endotoxin, transiently induces dysthymia, anhedonia and fatigue, i.e., symptoms which closely resemble core symptoms of MD (DellaGioia and Hannestad, 2010; Schedlowski et al., 2014). However, whether experimental endotoxemia also impacts neurocognitive processes relevant to the characteristic negative cognitions related to worthlessness, low self-esteem and hopelessness in MD remains unclear. Thus, we herein pooled data from randomized, double-blind, placebo-controlled endotoxin studies in order to assess endotoxin effects on self-esteem, hopelessness and the vulnerability factor intolerance of uncertainty. We observed the expected transient increases in circulating pro-inflammatory cytokines and body temperature, indicating systemic inflammation in response to low-dose LPS. Self-reported positive mood showed a transient decline after LPS application, which is in line with the well-established effects of LPS-induced systemic inflammation on mood (Reichenberg et al., 2001; Wright et al., 2005; Eisenberger et al., 2009; Hannestad et al., 2011; Benson et al., 2017a,b; Engler et al., 2017), and with previous reports documenting an association between dysthymia and LPS-induced increases in cytokine concentrations in plasma (Reichenberg et al., 2001; Eisenberger et al., 2010b) and cerebrospinal fluid (Engler et al., 2017).

Despite the clear and significant LPS-effect on mood, we did not find evidence that LPS induces low self-esteem, hopelessness, or an increased intolerance of uncertainty, i.e., negative thoughts which are common in MD. Interestingly, we observed that body-related self-esteem was slightly, but significantly lower in participants who received LPS when compared to the placebo group. This finding suggests that LPS-induced systemic inflammation may transiently impair certain aspects of self-esteem which are related to physical appearance, attractiveness and sportiness rather than affecting self-esteem in general. In detail, the respective subscale comprises items such as “*how often did you feel that other people are more athletically than you?*,” or “*how confident are you that other people think you are attractive?*” One likely explanation for the reported reduction in body-related self-esteem is that pro-inflammatory mediators released in response to LPS reportedly induce physical sickness symptoms such as fatigue and pain (Lekander et al., 2016; Benson et al., 2017b). Such symptoms conceivably impact self-perceived physical abilities, and hence the subscale of the questionnaire employed herein. Supporting this notion, we found that lower self-esteem ratings were associated with higher TNF-α concentration. Further, body-related self-esteem was significantly lowered in response to the higher LPS dose only, which mirrors the dose-dependent effects for cytokine concentrations and physical sickness symptoms (Wegner et al., 2014; Benson et al., 2017b). This could also indicate that only higher doses of LPS (and correspondingly higher cytokine concentrations) are capable to induce changes in body-related self-esteem. Moreover, LPS-induced systemic inflammation reportedly affects physical abilities such as walking speed (Sundelin et al., 2015), and even body odor (Olsson et al., 2014), alterations which can be detected by other persons (Regenbogen et al., 2017; Axelsson et al., 2018). Thus, lower ratings of body-related self-esteem may also be related to discrete, but perceivable changes in oneś own (outer) appearance during endotoxemia.

Taken together, our data suggest that although experimental endotoxemia effectively induces mood impairments, it does not alter self-referred negative cognitive schemata related to self-worth and hopelessness. Our finding of lower body-related self-esteem during systemic inflammation should be carefully interpreted in the light of its small effect size, which indicates that only a small proportion of the variance in body-related self-esteem was explained by the LPS application. This supports that additional factors including endotoxin-effects on physical sickness symptoms and physical abilities could have contributed to reduced body-related self-esteem. Moreover, it is also conceivable that the transient changes in body-related self-esteem are rather an adaptive and not a maladaptive response to an acute inflammatory event. Lowered body-related self-esteem may encourage reduced physical activity, and may thus contribute to saving energy resources which are needed during an immune activation (Straub, 2017).

Nevertheless, comprehensive evidence from independent studies and groups supports that the LPS model is well-suited to transiently induce other specific features of MD, which are related to mood impairments, social functioning, reward processing, reduced appetite, fatigue, increased pain sensitivity and unspecific physical symptoms (Schedlowski et al., 2014; Kiecolt-Glaser et al., 2015; Miller and Raison, 2016; Lasselin et al., 2018). With respect to cognitive symptoms of MD, it seems most likely that LPS affects cognitive *processes* such as the processing of emotional stimuli (Benson et al., 2017a), but not negative cognitive schemata, which are rather based on learning and repeated experiences (Beck et al., 1979; Bollen et al., 2017). This raises the question if self-referent negative cognitive schemata could be less responsive to inflammatory processes or pro-inflammatory mediators in general, i.e., if our finding might also apply for patients with MD. It is however also conceivable that negative cognitive schemata, while not responsive to a single and transient immune challenge, could be induced or worsened by repeated inflammatory events or chronic inflammation. Moreover, it is also possible that it is the *interaction* between inflammation and pre-existing vulnerability or situational factors such as psychological stressors, which ultimately affect cognitive schemata in persons at risk.

Our findings should be interpreted in the light of some limitations. Questionnaire data were not available from all primary studies, making it necessary to conduct the analyses in two separate data sets. Moreover, we did not measure cognitive symptoms at BL, making it impossible to control for intra-individual changes in response to LPS-administration. However, cognitive symptoms were assessed in all primary studies at similar time points in LPS- and placebo- groups under strictly standardized conditions. Thus, it seems unlikely that our findings are related to unsystematic differences between study groups. In addition, we can exclude anchor effects, i.e., biased responses due to a recall of BL ratings. We could herein assess only a limited number of inflammatory parameters. Recent research revealed that various peripheral and central cytokines, chemokines, and other molecules are involved in changes of cognitive functioning, including IL-4, Interferon-gamma, CCL-11 and β2-microglobulin (Villeda et al., 2011; Gadani et al., 2012; Smith et al., 2015; Monteiro et al., 2016). Future studies should take these parameters into account when addressing immune-related effects on cognitive functioning. Further, the LPS model allows to induce only a transient immune activation and we could include only young and healthy volunteers with BDI depression scores within a normal range. We therefore cannot conclude about possible associations between inflammatory parameters and self-esteem in individuals with a chronic immune activation and/or pre-existing mood symptoms, e.g., in clinically depressed patients. Thus, one important target for future research would be to assess the effects of immune parameters on negative cognitive schemata in persons at risk for psychiatric symptoms, or in elderly people, who are more prone to chronic inflammation (Zouikr and Karshikoff, 2017). Along the same lines, herein only a small number of female participants could be included in only one data set, which did not allow taking possible sex differences into account. Since only women using different types of hormonal contraceptives were included, it remains open if the results were confounded by different hormonal dosages, and can be translated to free-cycling women. Addressing sex differences and effects of hormonal status would be important given that women showed not only more pronounced responses to immune challenges (Klein, 2012), including LPS (Engler et al., 2016; Lasselin et al., 2018), but also greater LPS-induced changes in depressive symptoms (Moieni et al., 2015b), and a higher prevalence of MD (Hyde et al., 2008).

## Data Availability

The raw data supporting the conclusions of this manuscript will be made available by the authors, without undue reservation, to any qualified researcher.

## Author Contributions

SE, HE, MS and SB contributed to the conception and design of the study. TR, AW and AB collected data. SK and TR organized the database. SK and AB performed the statistical analysis and wrote the first draft of the manuscript. All authors contributed to manuscript revision, read and approved the submitted version. All authors agreed to be accountable for all aspects of the work in ensuring that questions related to the accuracy or integrity of any part of the work are appropriately investigated and resolved.

## Conflict of Interest Statement

The authors declare that the research was conducted in the absence of any commercial or financial relationships that could be construed as a potential conflict of interest. The reviewer SR declared their shared affiliation with the Editor.
